# Performance of China’s new medical licensing examination for rural general practice

**DOI:** 10.1186/s12909-020-02234-x

**Published:** 2020-09-17

**Authors:** Xinxin Han, Xiaotong Li, Liang Cheng, Zhuoqing Wu, Jiming Zhu

**Affiliations:** 1 School of Medicine, Tsinghua University, Beijing, China; 2 Institute for Hospital Management, Tsinghua Shenzhen International Graduate School, Shenzhen, China; 3 Department of Science and Education, Hainan Health Commission, Haikou, China; 4 Hainan Provincial Medical Association, Haikou, China; 5 Vanke School of Public Health, Tsinghua University, Beijing, 100084 PR China

**Keywords:** Medical licensing examination, Rural, General practice, Workforce supply, Medical education, Licensure

## Abstract

**Background:**

To evaluate the performance of China’s new medical licensing examination (MLE) for rural general practice, which determines the number of qualified doctors who can provide primary care for China’s rural residents, and to identify associated factors.

**Methods:**

Data came from all 547 examinees of the 2017 MLE for rural general practice in Hainan province, China. Overall pass rates of the MLE and pass rates of the MLE Step 1 practical skills examination and Step 2 written exam were examined. Chi-square tests and multivariable logistic regression were used to identify examinee characteristics associated with passing Step 1 and Step 2, respectively.

**Results:**

Of the 547 examinees, 68% passed Step 1, while only 23% of Step 1 passers passed Step 2, yielding an 15% (82 of 547) overall pass rate of the whole examination. Junior college medical graduates were 2.236 (95% CI, 1.127–4.435) times more likely to pass Step 1 than secondary school medical graduates. Other characteristics, including age, gender, forms of study and years of graduation, were also significantly associated with passing Step 1. In contrast, examinees’ vocational school major and Step 1 score were the only two significant predictors of passing Step 2.

**Conclusions:**

Our study reveals a low pass rate of China’s new MLE for rural general practice in Hainan province, indicating a relatively weak competency of graduates from China’s alternative medical education. An effective long-term solution might be to improve examinees’ clinical competency through mandating residency training for graduates of China’s alternative medical education.

## Background

Many countries have developed a medical licensing examination (MLE) system to determine and monitor the inflow of newly licensed doctors into the health workforce. In China, the MLE has been implemented for 20 years under the 1999 *Law on Practicing Doctors* [1]. The law requires that all practicing doctors in China must pass the MLE and register as a licensed doctor or a licensed assistant doctor in order to practice legally. These two cohorts of doctors actually represent graduates of China’s mainstream and alternative medical education pathways [2]. The mainstream pathway is standardized and takes the pattern of “5 + 3 + X”, that is, five-year undergraduate medical education followed by three-year residency training and X-year sub-specialty training. Graduates of the mainstream pathway can take the relevant MLE after working in a health organization for 1 year and register as a licensed doctor if passing the MLE. Accordingly, China’s licensed doctors are comparable to their international counterparts in high-income countries. China’s medical education system, especially this mainstream pathway and the standardised “5 + 3 + X” pattern, has been well documented in comparison with the patterns of the United Kingdom (UK) and the United States (US) [2], and generally the medical education system in China is more like the UK model.

However, China’s alternative medical education pathway is under-studied and much less familiar to the international audience. This alternative pathway, which often offers three-year medical education at the secondary school or junior college level, represents a historical legacy of the Soviet Union medical education modality. There is no nationally standardised curriculum for these instant programmes, and the three-year condensed curriculum often consists of one-year basic medical sciences, one-year clinical medicine and one-year clerkship [3, 4]. These education entities at the secondary school or junior college level are like sort of vocational training institutes in high-income countries. Graduates of the alternative pathway are also required to have one-year working experience before taking the relevant MLE and can register as a licensed *assistant* doctor after passing the MLE.

In 2018, China had about 3 million licensed doctors and 0.6 million licensed assistant doctors, but only 18% of them were working in rural primary care facilities (i.e., township health centres and village clinics^1^) [5]. Theoretically, rural primary care providers graduated from the alternative pathway are all supposed to pass the relevant MLE and register as licensed assistant doctors, but this transition has been very slow. In village clinics, particularly, below the qualification of licensed assistant doctors, for decades, China has maintained around 1 million “village doctors” who are de facto rural primary care providers [6]. Most village doctors are the historical legacy of the internationally famous “barefoot doctors” or graduates of the alternative education pathway. Some of them hold a village doctor certificate that allows them to prescribe and treat patients at village clinics with very limited scope of practice; the rest who do not have the certificate can only conduct the so-called public health tasks [7]. The low practicing qualification of many rural primary care providers is not only a challenge to the availability of qualified practicing doctors in rural China, but also a threat to the quality of care provided for rural residents.

In 2015, the central Chinese government added a new MLE for rural general practice into the existing MLE system to boost the number of qualified practicing doctors in rural areas [8]. The new MLE is designed specifically for providers who have worked in rural primary care facilities but not yet obtained the qualification of licensed assistant doctors. Successors of this MLE can register as rural general practitioners as part of the licensed assistant doctor group and are allowed to practice general medicine independently with full prescribing authorities at rural township health centres or village clinics. More importantly, this MLE provides greater incentives in retaining rural primary care providers. Successors can obtain a headcount (called *Bianzhi* [9] in Chinese), a tenure position that is considered as formal employees of China’s public sectors, often with higher salaries, secured pensions and other benefits.

The MLE for rural general practice was piloted in nine provinces in 2016, then further expanded to another 15 provinces in 2017, and finally implemented nationwide in 2018. Consistent with the existing MLEs, this MLE also consists of two steps, but its content is tailored more to general practice in rural settings. The Step 1 examination is a five-station practical skills test, and the five stations consist of medical history taking, physical examinations, basic clinical operations, basic public health operations, and basic traditional Chinese medicine (TCM) operations [10]. Step 2 is a one-day written examination, adopting the format of multiple-choice questions (300 in total) and assessing examinee’s knowledge about general medicine, public health, ethics, and healthcare regulations [11]. Examinees must pass both Step 1 and Step 2 to succeed in the whole MLE.

Understanding the performance of this MLE for rural general practice is vital, as it determines the number of qualified practicing doctors who can provide primary care for China’s rural residents. China has set the goal that by 2020, the majority of village doctors should obtain the qualification of licensed assistant doctors [12]. However, evidence regarding the performance of this MLE is very limited. To our knowledge, only two domestic analyses examined the performance of this MLE across the nine piloted provinces in 2016 [13, 14]. These descriptive analyses found a relatively higher pass rate in Step 1 (68%) and a lower rate in Step 2 (55%), yielding an overall 31% pass rate of the whole MLE. One of these analyses also showed that Step 1 pass rate varied by gender, ethnicity, education level, years of graduation and major. Nonetheless, evidence is still lacking on subgroup performance differences in Step 2, as well as the performance in other provinces after this MLE was expanded.

In this study, we aimed to extend the literature by examining a broader range of examinee characteristics and their association with passing Step 1 and Step 2, respectively, using data from all examinees of the MLE for rural general practice in 2017 in Hainan province, one of the provinces that started this MLE in 2017. As increasing the supply of qualified primary care providers, particularly in underserved areas, is a worldwide challenge regardless of economic status, we hope that studying China’s situation, particularly from the MLE and medical education aspects, can provide useful insights for those tackling similar challenges in other parts of the world.

## Methods

### Data source and study population

This study used data from Hainan MLE administrative database, provided by Hainan Provincial Medical Association. This database contains information for all examinees of the 2017 MLE in Hainan, including gender, date of birth, major, forms of study, educational level, graduation school, graduation year, practice location, type of examination that was taken and examination score.

All 547 examinees of the MLE for rural general practice in 2017 in Hainan were included in this analysis.

### Study outcome

The outcome variables were a “pass” or “fail” on Step 1 and Step 2, respectively. The cut-off passing score was 60 for Step 1 and 180 for Step 2. For analysis, examinees who received a score 60 and above in Step 1 was assigned a value of 1 = pass, otherwise 0. Because an examinee must pass Step 1 before taking Step 2, we created another dummy variable for Step 1 passers who received a score 180 and above in Step 2.

### Examinee characteristics

We examined examinee characteristics that are available in the database, including age (age below 35, age between 35 and 45, vs age above 45), gender, vocational school major (western medicine, traditional Chinese medicine or combination of western and Chinese medicine, and other specialties including rural medicine, community medicine or healthcare), educational level (secondary school vs junior college level medical education), years of graduation (graduated from school less than 10 years, 10–20 years, vs above 20 years), forms of study (full-time vs part-time), practice facility (township health centre vs village clinic), in-province graduates (i.e., graduated from schools in Hainan), and whether having a village doctor certificate.

### Statistical analysis

We calculated the pass rates for Step 1 and Step 2, respectively, and compared pass rates by examinee characteristics, using Pearson Chi-square tests. We then constructed multivariable logistic regression models to examine predictors of passing Step 1 and Step 2, respectively. Models were run separately for those who took Step 1 and those who took Step 2 after passing Step 1. In the model testing Step 2 success, we also added a categorical variable of Step 1 score in addition to examinee characteristics. Step 1 score was reclassified by quartile using the percentile rank for each score. We clustered standard errors by examinee’s practice facility to adjust autocorrelation among examinees practicing in the same facility. Adjusted odds ratios (AOR) and 95% confidence intervals (CI) from regression analysis were presented. An alpha level of 0.05 was used to determine variable significance. All analyses were performed using Stata 15 (StataCorp).

## Results

### Examinee characteristics

Table 1 presents the characteristics of 547 examinees of the MLE for rural general practice in Hainan in 2017. The majority of examinees were male (74%), aged above 35 (77%), received secondary school medical education (84%), graduated 10 years or more (64%), and took the form of full-time study (82%). About 30% majored in western medicine, 25% in traditional Chinese medicine or combination of western and Chinese medicine, and the rest of 45% majored in rural medicine or other areas. Two thirds (61%) graduated from Hainan. Less than one-third had a village doctor certificate (23%). Less than one-third (26%) worked in village clinics and the rest (74%) worked in township health centres.

**Table 1:** Examinee characteristics of china’s medical licensing examination for rural general practice in Hainan Province, 2017

Characteristics	No.	%
**Total No.**	**547**	**100**
**Age**		
< 35	126	23
35–45	292	53
> 45	129	24
**Gender**		
Male	407	74
Female	140	26
**Vocational School Major**		
Western medicine	165	30
Chinese medicine or combination of western and Chinese medicine	134	25
Rural medicine, community medicine or healthcare	248	45
**Educational Level**		
Secondary school	458	84
Junior college	89	16
**Years of Graduation**		
< 10 years	198	36
10–20 years	289	53
> 20 years	60	11
**In-Province Graduate**		
No	211	39
Yes	336	61
**Forms of Study**		
Part-time	98	18
Full-time	449	82
**Having a Village Doctor Certificate**		
No	423	77
Yes	124	23
**Practice Facility**		
Village clinic	142	26
Township health centre	405	74

### Overall pass rates

Table 2 presents the overall pass rate and pass rates of the MLE Step 1 practical skills examination and Step 2 written examination in Hainan. In 2017, 357 (65%) of the 547 examinees passed the Step 1, and among these passers, 82 (23%) passed Step 2, yielding an overall 15% (82 out of the 547) pass rate for the entire examination.

**Table 2:** Overall pass rates of china’s medical licensing examination for rural general practice in Hainan Province, 2017

	Candidates, No.	Passers, No.	Pass Rate, %
Step 1 Practical Skills Examination	547	357	65
Step 2 Written Examination	357	82	23
Overall	547	82	15

### Pass rates by examinee characteristics

Table 3 presents subgroup performance of the MLE Step 1 and Step 2. The bivariate analysis shows that in the Step 1, pass rates vary significantly by age, gender, educational level, and forms of study. The youngest group (age < 35) had the highest pass rate (73%), followed by the middle-age group (age between 35 and 45, 67%), and those aged above 45 had the lowest pass rate (54%, *p* = 0.005). The pass rates were significantly higher among male examinees versus female examinees (68% vs 56%, *p* = 0.011), among junior college medical graduates versus secondary school medical graduates (75% vs 63%, *p* = 0.030), and among those studied full-time in school versus those studied part-time (67% vs. 56%, *p* = 0.036).

**Table 3:** Bivariate analysis of step 1 and step 2 pass rates by examinee characteristics

	Pass Rates, %			
	Step 1 Practical Skills Examination (***N*** = 547)	*P* value	Step 2 Written Examination (***N*** = 357)	*P* value
**Age**				
< 35	73	0.005	27	0.132
35–45	67		24	
> 45	54		14	
**Gender**				
Male	68	0.011	22	0.574
Female	56		25	
**Vocational School Major**				
Western medicine	67	0.843	28	< 0.001
Chinese medicine or combination of western and Chinese medicine	63		35	
Rural medicine, community medicine or healthcare	65		13	
**Educational Level**				
Secondary school	63	0.030	22	0.245
Junior college	75		28	
**Years of Graduation**				
< 10 years	60	0.107	21	0.854
10–20 years	68		24	
> 20 years	70		24	
**In-Province Graduate**				
No	68	0.246	22	0.594
Yes	63		24	
**Forms of Study**				
Part-time	56	0.036	24	0.898
Full-time	67		23	
**Having a Village Doctor Certificate**				
No	67	0.204	24	0.492
Yes	61		20	
**Practice Facility**				
Village clinic	59	0.076	25	0.613
Township health centre	67		22	

*P* values were from Pearson Chi-square tests

In Step 2, pass rates vary significantly only by major. Examinees majored in traditional Chinese medicine or combination of western and Chinese medicine had the highest pass rate (35%), followed by those majored in western medicine (28%), and those majored in rural medicine or other areas had the lowest pass rate (13%, *p* < .001).

### Characteristics associated with passing step 1 and 2

Table 4 presents the regression analysis results of characteristics associated with passing Step 1 and Step 2. Junior college graduates were 2.236 (95% CI = 1.127–4.435) times more likely to pass Step 1 than those secondary school graduates. Examinees graduated more than 20 years were 2.396 (95% CI = 1.208–4.751) times more likely to pass Step 1 than those graduated less than 10 years. Examinees who studied full-time in school were 1.722 (95% CI = 1.021–2.905) times more likely to pass Step 1 than those who studied part-time. Examinees aged above 45 were 0.354 (95% CI = 0.195–0.641) times less likely to pass Step 1 compared to those aged below 35. Compared to female examinees, male examinees were 1.611 (95% CI = 1.056–2.458) times more likely to pass Step 1.

**Table 4:** Multivariable logistic regression analysis of examinee characteristics associated with passing step 1 and step 2

Variables	Step 1 Practical Skills Examination		Step 2 Written Examination	
	AOR (95% CI)	***P*** Value	AOR (95% CI)	***P*** Value
**Age**				
< 35	Reference		Reference	
35–45	0.709 (0.426–1.180)	0.186	1.108 (0.556–2.209)	0.770
> 45	0.354 (0.195–0.641)	0.001	0.444 (0.183–1.073)	0.071
**Gender**				
Female	Reference		Reference	
Male	1.611 (1.056–2.458)	0.027	1.024 (0.521–2.010)	0.945
**Vocational School Major**				
Western medicine	Reference		Reference	
Chinese medicine or combination of western and Chinese medicine	1.177 (0.658–2.106)	0.582	1.306 (0.596–2.864)	0.505
Rural medicine, community medicine or healthcare	1.507 (0.884–2.569)	0.132	0.382 (0.175–0.837)	0.016
**Educational Level**				
Secondary school	Reference		Reference	
Junior college	2.236 (1.127–4.435)	0.021	1.033 (0.175–0.837)	0.938
**Year of Graduation**				
< 10 years	Reference		Reference	
10–20 years	1.399 (0.908–2.156)	0.128	0.846 (0.175–0.837)	0.612
> 20 years	2.396 (1.208–4.751)	0.012	1.378 (0.175–0.837)	0.566
**In-Province Graduate**				
No	Reference		Reference	
Yes	0.726 (0.483–1.091)	0.124	1.059 (0.175–0.837)	0.851
**Forms of Study**				
Part-time	Reference		Reference	
Full-time	1.722 (1.021–2.905)	0.042	1.126 (0.465–2.726)	0.793
**Having a Village Doctor Certificate**				
No	Reference		Reference	
Yes	0.973 (0.624–1.515)	0.902	0.781 (0.465–2.726)	0.495
**Practice Facility**				
Village clinic	Reference		Reference	
Township health center	1.257 (0.822–1.923)	0.291	1.026 (0.536–1.964)	0.939
**Step 1 Score**				
Quartile 1, < 61.5			Reference	
Quartile 2, 61.5–67.5			1.767 (0.741–4.213)	0.199
Quartile 3, 68–73.5			2.307 (1.000–5.326)	0.050
Quartile 4, > 73.5			4.137 (1.797–9.526)	0.001
Adjusted R^2^	0.055		0.098	
Observations	547		357	

Standard errors were clustered at the practice facility level

Among examinees who passed Step 1, examinees majored in rural medicine or other areas were less likely to pass Step 2 compared to those majored in western medicine (AOR = 0.382, 95% CI = 0.175–0.837), while there was no significant difference in passing Step 2 between those majored in western medicine and Chinese medicine. In addition, Step 1 score positively predicts the Step 2 success. Compared to examinees with a score in the lowest quartile, those with a score in quartile 4 were the mostly likely to pass the written examination (AOR = 4.137, 95% CI = 1.797–9.526), followed by those who had a score in quartile 3 (AOR = 2.307, 95% CI = 1.000–5.326).

## Discussion

China has launched a new MLE for rural general practice to increase the number of qualified practicing doctors in rural China. Our analysis presents new evidence on the performance of this MLE, as well as examinee characteristics associated with passing the Step 1 practical skills examination and the Step 2 written examination. Findings of this study, coupled with prior results [10, 11], indicate a low overall pass rate of this new MLE, suggesting that this new MLE has not yet met its expectation of increasing qualified doctor supply in rural China. Although more than two-thirds of examinees passed Step 1, the clinical knowledge of examinees is insufficient to pass Step 2. We found that age, gender, educational level, and years of graduation were significantly associated with passing Step 1. Our analysis added new evidence that examinee’s vocational school major and Step 1 score were the only two significant predictors of passing Step 2.

The low pass rate of this MLE for rural general practice reflects a weak competency of graduates from China’s alternative medical education. As a legacy of the Soviet Union education model, the alternative education has not yet been standardised and may create many challenges to quality of care. This alternative pathway even consists of two levels of education: the secondary school level education and the junior college level education, which offers a 3-year medical training that emphasizes on a low level of clinical competency and little on systematic clinical knowledge. Our findings support the abolishment of the secondary school medical education [15]. More importantly, the secondary school medical education may offer programmes of rural medicine or community medicine, in addition to regular medicine [16, 17]. These programmes reflect the low-level overspecialisation at a too early stage in the Soviet Union model. We found that examinees majored in rural medicine or community medicine were less likely to pass the final written examination compared to those majored in general western medicine, suggesting the misappropriation of offering specialisations in the secondary school level education. In fact, this overspecialisation is the only significant predictor of failing in the final step examination, in addition to the Step 1 score. Offering rural medicine or community medicine in the ‘residency’ stage may be more effective in building up the capacity needed in practice.

Although the 5-year undergraduate medical education is now the mainstream medical education in China, until 2018, graduates of the alternative pathway still represent more than one-third of China’s doctor group [5]. There have been concerns on the quality of doctors produced by the alternative education, as it provides a legitimate entry into the medical profession without receiving standardized and systematic medical education, leading to large variations in the competency of practicing doctors and poor performance in the MLEs. In fact, there has been evidence demonstrating poor care quality in China’s rural clinics [18], at least partially attributed to the poor competency of village doctors [19]. Since there is currently no evidence of the relationship between passing this MLE and quality of care in China, future research is still warranted to investigate this relationship.

Our results highlight the need for improving the performance of examinees in this MLE, especially the performance in Step 2. A short-term solution might be to provide additional training to examinees before taking the MLE. In 2013, China implemented a National Rural Doctor Education Plan (2010–2020) [12], urging local governments to offer exam-targeted intensive training to village doctors who have not yet obtained the qualification of licensed doctors or assistant doctors. This training is offered once every 3–5 years with training time less than 1 month and emphasizes clinical diagnosis and treatment, public health services, and special skills. A prior study has revealed that village doctors view this exam-targeted training is helpful for passing the MLE [20].

However, such a short-term training may not be sufficient to help examinees to reach the exam requirements. Our results of low performance in Step 2 suggest that a more theory-based education such as review courses before the exam might be more helpful for the examinees to succeed in the MLE. There has been evidence in the US showing that review course helps improve examinees’ USMLE Step 1 score [21]. Prior survey studies also revealed a high demand of training on medical knowledge among China’s rural primary care providers [22, 23]. Such an effort could be made greater to examinees who majored in rural medicine or community medicine, as our results show that they were less likely to pass Step 2 than those majored in western medicine or Chinese medicine.

In a long-term, a more effective solution might be to reform the ‘residency’ stage for graduates of the alternative education and further improve their clinical competency. Our study shows that higher Step 1 score is a strong predictor of passing Step 2, suggesting a potential connection between clinical competency and clinical knowledge. Since 2012, China has offered a two-year residency training for secondary school or junior college graduates, but this requirement is voluntary [24]. For the mainstream education, China has already made the three-year residency training compulsory nationwide [25]. There were also numerous studies demonstrating the role of residency training in improving the clinical capacity of practitioners in other countries [26–28]. Therefore, it is imperative to consider making the residency training mandatory for graduates of the alternative education. To the extent that training improves the competency of China’s practicing doctors, especially the practicing doctors in rural areas, it is vital to continue tracking and monitoring the performance of the MLE.

We acknowledged several limitations of this study. First, our analysis was limited by the data we used. In the analysis, we used data from only one province, so the results may not be generalized to the rest parts of China. It is important for future research to revisit this study and examine the performance of the MLE at the national level. Yet, our results regarding the MLE Step 1 are consistent with prior findings that examined the performance of this MLE in the nine piloted provinces in 2016, suggesting the validity of our results. Second, although we included all examinees of the MLE for rural general practice in Hainan in the analysis, the small number of observations may limit the statistical power to detect statistical significance. Lastly, due to the cross-sectional design of this study, our results cannot infer causality.

## Conclusions

Our study finds that the pass rate of the MLE for rural general practice in Hainan in 2017 was low and that examinee characteristics were more related to passing Step 1 but not Step 2. On the other hand, the low pass rate indicates that this new examination still preserves the basic standard of becoming a licensed assistant doctor in China. This is consistent with the principle of setting up the MLE – to maintain safe practice and patients’ confidence in doctors. Now at a critical stage of its efforts to increase the availability of qualified primary care providers in rural areas, China needs to strike a balance between the quantity and quality of newly licensed assistant doctors. Many other countries may also have to deal with this dilemma. As China mandates residency training for mainstream medical education graduates, residency training should also be reformed and mandated to graduates from the alternative pathway in order to improve their performance both in the MLE and in practice. More research is still needed to understand the role of the MLE in ensuring quality of care provided for China’s rural residents.

## Data Availability

The data that support the findings of this study are available from Hainan Provincial Medical Association but restrictions apply to the availability of these data, which were used under license for the current study, and so are not publicly available.

## Abbreviation

MLE: Medical licensing examination

## Footnotes

1 China has a world-famous three-tier rural healthcare system. The first tier is village clinics – these rural outpatient clinics provide primary health care. The second tier is township health centres, many of which have inpatient beds and are like small community hospitals. They may also provide technical and workforce support to village clinics. The third tier, which is at the top of the rural healthcare system, is county hospitals that are often public hospitals with relatively big outpatient and inpatient departments. These village clinics and township health centres are China’s rural primary care facilities, where all the examinees in this article are working.
